# Improving beef hamburger quality and fatty acid profiles through dietary manipulation and exploitation of fat depot heterogeneity

**DOI:** 10.1186/2049-1891-5-54

**Published:** 2014-11-24

**Authors:** Cletos Mapiye, Jennifer L Aalhus, Payam Vahmani, David C Rolland, Timothy A McAllister, Hushton C Block, Bethany Uttaro, Spencer D Proctor, Michael E R Dugan

**Affiliations:** Agriculture and Agri-Food Canada, Lacombe Research Centre, 6000 C and E Trail, Lacombe, Alberta T4L 1 W1 Canada; Department of Animal Sciences, Faculty of AgriSciences, Stellenbosch University, P. Bag X1, Matieland, 7602 South Africa; Agriculture and Agri-Food Canada, Lethbridge Research Centre, 1st Avenue South 5403, PO Box 3000, Lethbridge, Alberta T1J 4B1 Canada; Metabolic and Cardiovascular Diseases Laboratory, Diabetes and Mazankowski Institutes, Li Ka Shing Centre for Health Research Innovation, University of Alberta, Edmonton, Alberta T6G 2E1 Canada

**Keywords:** Beef, DDGS, Fat depot, Fatty acids, Sensory attributes, Oxidative stability

## Abstract

**Background:**

Hamburger is the most consumed beef product in North America, but lacks in nutritional appeal due to its high fat content and high proportion of saturated fatty acids (SFA). Objectives of the present study were to improve the FA profiles of hamburgers made with perirenal fat (PRF) and subcutaneous fat (SCF) when feeding steers different diets along with examining differences in sensory attributes and oxidative stability. Diets included a control diet containing 70:30 red clover silage: barley based concentrate, a diet containing sunflower-seed (SS) substituted for barley, and diets containing SS with 15% wheat dried distillers’ grain with solubles (DDGS-15) or 30% DDGS (DDGS-30). Hamburgers were made from *triceps brachii* and either PRF or SCF (80:20 w/w).

**Results:**

Perirenal fat versus SCF hamburgers FA had 14.3% more (*P* <0.05) 18:0, 11.8% less *cis* (*c)*9-18:1 (*P* <0.05), and 1.82% more total *trans* (*t*)-18:1 mainly in the form of *t*11-18:1. During sensory evaluation, PRF versus SCF hamburgers had greater (*P* <0.05) mouth coating, but the difference was less than one panel unit. Examining effects of steer diet within PRF hamburgers, feeding the SS compared to the control diet increased (*P* <0.05) *t*-18:1 by 2.89% mainly in the form of *t*11-18:1, feeding DGGS-15 diet led to no further changes (*P* >0.05), but feeding DDGS-30 diet reduced the proportions of (*P* <0.05) of *t*-18:1 chiefly *t*11-18:1. Feeding SS and DDGS diets had small but significant (*P* <0.05) effects on hamburger sensory attributes and oxidative stability.

**Conclusions:**

Feeding high-forage diets including SS and 15% DDGS, and taking advantage of the FA heterogeneity between fat depots offers an opportunity to differentially enhance beef hamburgers with 18:2*n*-6 biohydrogenation products (*i.e., t*11-18:1) with potential human health benefits without compromising their sensory attributes and oxidative stability during retail display.

## Background

Ground beef and its by-products, including hamburger containing up to 30% added fat, are the most commonly purchased beef products in North America [1], probably due to their price and preparatory versatility. Of the 28.5 kg/capita per annum consumption of fresh beef in North America [2], about 52% is ground beef [3]. However, the consumer perception of the healthfulness of beef, especially ground beef and hamburgers has been declining [3], largely because it is a rich source of saturated fatty acids (SFA) that have relationships with several diseases from cardiovascular disease (CVD) to cancer [4]. On the other hand, beef contains polyunsaturated fatty acids (PUFA) biohydrogenation products (BHP) including rumenic acid (*t*9,*c*11-18:2) and its precursor vaccenic acid (*t*11-18:1), which may have potential human health benefits [5]. In this regard, enriching beef and its further processed products with unsaturated fatty acids (FA) is one strategy that could be used to gain consumer confidence and subsequently improve the image of beef.

Hamburger can be enriched with unsaturated FA through animal nutrition with the strategic use of forages and dietary lipids [6], and by the selective use of trim fats which can contain different amounts of PUFA-BHP [7]. Feeding cattle high-forage diets with sunflower-seed (*i.e.,* a rich source of linoleic acid, 18:2*n*-6) is an effective way to promote deposition of *t*11-18:1 and *t*9, *c*11-18:2, with greater accumulations of total PUFA-BHP in perirenal fat (PRF) *vs.* subcutaneous fat (SCF) [8]. With these diets, however, the high forage content (up to 70% DM) negatively affects animal performance and beef quality (*i.e.,* marbling fat deposition) [9]. In a follow-up experiment we examined the effects of substituting red clover silage with a non-forage fibre source (wheat dried distiller’s grains plus solubles (DDGS)) along with sunflower seed (SS) addition to the diet and found that it improved beef quality while maintaining or increasing PUFA-BHP proportions in beef [10]. Wheat DDGS has been found to have a feeding value comparable to barley grain while maintaining beef quality [11], and has been shown to increase *t*9,*c*11-18:2 and *t*11-18:1 [12], while reducing *t*10-18:1 which has been shown to negatively affect blood lipid profiles in animal models [13]. The objectives of the present study were to examine hamburger quality and FA composition from this trial, first comparing hamburgers made with SCF *vs.* PRF, and then to further investigate specific effects of diet on PRF hamburger. Our focus was on PRF as it may be an underutilized fat depot that is easily accessible during the slaughter process. It has a higher content of total PUFA-BHP, but has a higher SFA content *vs.* SCF [8]. The difference in SFA is, however, almost entirely due to an increased amount of 18:0, which is known to have neutral effect on plasma cholesterol profiles when consumed by humans [14].

## Material and methods

### Animals and diets

Procedures for the care and handling of animals used in this study were approved by the Lacombe Research Centre Animal Care Committee in compliance with the principles and guidelines established by the Canadian Council on Animal Care [15]. Tissues used for the current study were collected from the study reported by Mapiye et al. [10]. In summary, 64 12-month-old British × Continental crossbred steers with an initial mean body weight (BW) of 362.7 ± 4.50 kg were stratified by weight to four experimental diets (control, SS, DDGS-15 and DDGS-30), with two pens of eight steers/diet. The control diet was composed of 70% red clover silage, 25.8% barley grain and 4.2% vitamin*-*mineral supplement on a dry matter (DM) basis (Table 1). The SS diet contained 11.4% SS substituted for barley grain, and the DDGS-15 and DDGS-30 diets contained 15 and 30% DDGS substituted for red clover silage and SS to maintain a targeted 5% added oil in the diets from either SS or DDGS (DM basis; Table 1).

**Table 1:** **Ingredient, nutrient and fatty acid composition of the dietary treatments**

Variable	Dietary treatments				
	Control	SS	DDGS-15	DDGS-30	
Ingredients, % DM basis					
Sunflower-seed	0.0	11.4	9.2	7.0	
Dried distiller’ grains with solubles	0.0	0.0	15.0	30.0	
Barley grain	25.8	14.4	14.4	14.4	
Red Clover	70.0	70.0	57.2	44.4	
Vitamin/mineral supplement^1^	4.2	4.2	4.2	4.2	
Nutrient composition, % DM basis					SD
Dry matter	42.6	40.2	44.2	50.1	4.2
Crude protein	13.1	13.4	16.5	20.8	3.6
Crude fat	1.89	6.40	5.80	5.90	2.09
Calcium	0.86	0.92	0.81	0.69	0.10
Phosphorus	0.31	0.32	0.41	0.53	0.10
Acid detergent fibre	33.7	37.0	33.8	28.4	3.6
Neutral detergent fibre	43.3	48.7	44.5	38.5	4.2
Digestible Energy, Mcal/kg	2.71	2.57	2.73	2.91	0.14
Fatty acids, % of total fatty acids					
14:0	0.35	0.17	0.15	0.15	0.10
16:0	18.8	10.6	11.9	13.5	3.60
18:0	2.86	4.15	3.67	3.24	0.56
20:0	1.11	0.65	0.49	0.41	0.31
22:0	1.29	1.11	0.85	0.71	0.26
24:0	1.25	0.70	0.52	0.44	0.36
*c*9-18:1	9.49	12.4	13.0	13.3	1.75
*c*11-18:1	0.93	0.73	0.76	0.78	0.09
18:3*n*-3	18.9	7.09	6.26	5.59	6.32
18:2*n*-6	39.0	59.6	60.3	60.1	10.5

SS, sunflower-seed; DDGS-15; 15% wheat dried distillers’ grain with solubles + sunflower-seed; DDGS-30, 30% wheat dried distillers’ grain with solubles + sunflower-seed; SD, standard deviation; ^1^Vitamin/mineral supplement/kg DM contained 1.86% calcium, 0.93% phosphorous, 0.56% potassium, 0.21% sulphur, 0.33% magnesium 0.92% sodium, 265 ppm iron, 314 ppm manganese, 156 ppm copper, 517 ppm zinc, 10.05 ppm iodine, 5.04 ppm cobalt, 2.98 ppm selenium, 49,722 IU/kg vitamin A, 9,944 IU/kg vitamin D3, and 3,222 IU/kg vitamin E.

### Sample collection and preparation

At slaughter, PRF was collected approximately 30 min post-mortem, vacuum packed and held in a 2°C cooler with a wind speed of 0.5 m/s. Carcasses were cooled for 24 h under the same conditions prior to collection and vacuum packing of *triceps brachii* muscle and SCF from along the dorsal region, over the *longissimus thoracis et lumborum*. After 6 d storage, 80:20 (w/w) lean:fat hamburger was prepared with an initial grind through a 6 mm plate, then mixed and ground through a 4 mm plate (Butcher Boy meat grinder Model TCA22, Lasar Manufacturing Co, Los Angeles, CA, USA). A 50 g subsample of each grind was collected for an immediate 0 h thiobarbituric acid reactive (TBAR) substance determination [16]. A second 50 g sample was blended using a Robot Coupe Blixir BX3 food processor (Robot Coupe USA Inc., Ridgeland, MS, USA) and frozen at -80°C for subsequent FA analysis. Three 140 g hamburger patties (11.4 cm diameter × 0.63 cm thick) were formed from remaining grind using a single hamburger press (Cabelas, Sydney, NE, USA). One patty was placed on a polystyrene tray, over-wrapped with an oxygen permeable polyvinylchloride film (oxygen transmission rate 8,000 mL/m^2^/24 h; Vitafilm Choice Wrap, Goodyear Canada Inc.) and placed into a fan assisted, horizontal retail display case (Hill Refrigeration of Canada Ltd., Barrie, ON, Canada) with an average temperature of 3.5°C for objective colour measurements at 0 and 4 d. Samples were held under fluorescent room lighting (GE deluxe cool white; General Electric Canada, Oakville, ON, Canada) supplemented with incandescent lighting directly above the display case (GE clear cool beam 150 W/120 V; General Electric Canada) spaced 91.5 cm apart to provide an intensity of 1,076 lux at the meat surface for 12 h per d. After 4 d in retail display, following objective colour measurements, TBAR determination was performed on the patty. The other two remaining patties/animal/depot were vacuum packaged and stored at -20°C for subsequent sensory evaluation.

### Colour measurements

Objective colour measurements on hamburger patties were performed at three separate locations over the patty surface using a Minolta CR-300 with Spectra QC-300 Software (Minolta Canada Inc., Mississauga, ON, CA). Three colour measurements, L*, a* and b* values [17] were used to calculate hue = tan^-1^(b*/a*); and chroma = (a*^2^ + b*^2^)^0.5^. Spectral reflectance readings were also collected at the same time and converted to reflex attenuance [18]. Interpolation of isobestic points for 473, 525, 572, and 700 nm were determined to calculate relative contents of metmyoglobin and oxymyoglobin [19]. Retail display effects on L*, hue, chroma, metmyoglobin and oxymyoglobin were calculated as the difference between 0 d and 4 d values.

### Fatty acid analysis

Hamburger samples were thawed and lipids extracted with 2:1 chloroform:methanol according to Folch et al. [20]. Separate 1.5 mol/L methanolic HCl and 0.5 mol/L sodium methoxide methylations of hamburger lipid extracts (10 mg) were performed according to Kramer et al. [21] with the inclusion of 1 mg *c*10-17:1 (Nu-Chek Prep. Inc. Elysian, MN, USA) as an internal standard. Fatty acid methyl esters were analysed by GC using a CP-Sil88 column (100 m, 25 μm ID, 0.2 μm film thickness, Varian Inc., Walnut Creek, CA, USA) using complementary temperature programs with 150°C and 175°C plateaus according to Kramer et al. [21]. Conjugated linoleic acid (CLA) isomers not separated by GC were further analysed using Ag^+^-HPLC as described by Cruz-Hernandez et al. [22]. Individual peaks were identified using reference standards (GLC-603, Nu-Chek Prep. Inc., Elysian, MN, USA; BC-Mix1, Applied Science, State College, PA, USA) and peak order and retention times reported in the literature [21–23]. Only groups/families of FA and major FA within groups were reported.

### Sensory evaluation

Hamburger patties were placed on a tray in a single layer, and thawed overnight at 4°C. Hamburgers were weighed and then cooked in individual non-stick pans on an electric grill (Garland Grill ED30B, Condon Barr Food Equipment Ltd., Edmonton, AB) pre-heated to 205°C. Previous testing with surplus prepared patties indicated hamburger patties reached an internal temperature of 71°C with juices running clear after cooking for four min on one side, flipping and then cooking an additional eight min. Following cooking in this manner, hamburger patties were cooled for two min prior to recording final weights and calculating cooking loss. Hamburger patties were divided into eight equal wedges and presented to eight panellists trained according to the American Meat Science Association [24] research guidelines. Panellists evaluated six samples/session and attended four sessions/d with the experimental treatments randomized amongst these sessions. Attribute ratings were electronically collected (Compusense Inc., Guelph, On, Canada) using nine point descriptive scale for initial and overall tenderness (9 = extremely tender; 1 = extremely tough), initial and sustainable juiciness (9 = extremely juicy; 1 = extremely dry), flavour and off-flavour intensity (9 = extremely intense; 1 = extremely bland/none), and residual mouth coating (9 = no mouth coating; 1 = extreme mouth coating).

### Statistical analysis

Statistical analyses were conducted using Proc Mixed SAS [25]. To determine the main effect of fat depot location, the following statistical model was used: Y_ijk_ = μ + α_i_ + β_j_ + αβ_ij_ + ϵ_ijk;_ where Y_ijk_ is the observation (fatty acids, sensory scores, colour and lipid oxidation values), μ is the overall mean, α_i_ is the effect of the i^th^ fat depot (i = SCF, PRF; df = 1), β_j_ is the effect of j^th^ diet (j = control, SS, DDGS-15, DDGS-30; df = 2), αβ_ij_ is the effect of the interaction between fat depot and diet and ϵ_ijk_ (df = 2) is the residual error (df = 58). Slaughter day and animal within a diet were incorporated as random effects. A data sub-set of the PRF hamburgers was then analysed to determine the effects of diet using the following statistical model: Y_ij_ = μ + α_i+_ ϵ_ij,_ where Y_ij_ is the observation, μ is the overall mean, α_i_ is the effect of the i^th^ diet (df = 2) and ϵ_ij_ is the residual error (df = 61). Slaughter day and animal were included as random effects. Means were generated and separated using the LSMEANS and PDIFF options, respectively. The threshold for significance was set at *P* <0.05.

## Results and discussion

### Animal performance and meat quality

Animal performance and meat quality results were detailed by Mapiye et al. [10]. In summary, steers fed the DDGS-30 diet tended (*P* = 0.10) to have the highest DMI, followed by steers fed DDGS-15, control and SS diets during the course of the experiment. These results were related to the fibre content reported for the respective diets. Steers fed the DDGS-30 diet had the greatest ADG, final weight and cold carcass weight followed by steers fed the DDGS-15, control and SS diets (*P* <0.05). These results were related to relatively higher digestible energy and a trend for increasing DMI reported for the DDGS containing diets. Meat from steers fed the SS and control diets had similar (*P* >0.05) grade fat, rib eye area, lightness and sensory panel meat tenderness. However, meat from steers fed DDGS diets had greater (*P* <0.05) grade fat, rib eye area, lighter (*P* <0.05) colour and improved (*P* <0.05) instrumental and sensory panel meat tenderness relative to steers fed the SS diet. These results are a reflection of the growth performance results reported for the respective diets.

### Fatty acid composition of hamburgers made with perirenal and subcutaneous fats

#### Fatty acids of endogenous or dietary origin

Perirenal fat hamburgers had higher (*P* <0.05) total FA content than SCF hamburgers (Table 2). These findings are likely associated with differences in moisture between PRF and SCF, with SCF typically containing more water than PRF [26].

**Table 2:** **Fatty acid composition of hamburgers (% of total fatty acids) made with perirenal or subcutaneous fat across all diets**

	Fat depot location		s.e.m	***P*** -value
Variable	Perirenal (n = 64)	Subcutaneous (n = 64)		
∑ FA, mg/g	194.5^a^	189.3^b^	0.9	0.02
FA of endogenous or dietary origin				
∑ SFA	55.6^a^	40.3^b^	0.4	<0.001
14:0	2.96^a^	2.80^b^	0.11	0.01
16:0	24.7^a^	24.1^b^	0.2	<0.001
18:0	27.5^a^	13.2^b^	0.3	<0.001
∑ *c*-MUFA	30.0^b^	44.5^a^	0.3	<0.001
*c*9-14:1	0.33^b^	1.07^a^	0.04	<0.001
*c*9-16:1	1.45^b^	4.33^a^	0.07	<0.001
*c*9-18:1	23.5^b^	35.3^a^	0.2	<0.001
∑ *n*-3	0.47	0.48	0.01	0.47
18:3*n*-3	0.39	0.38	0.01	0.07
22:5*n*-3	0.09^b^	0.10^a^	0.00	0.01
∑ *n*-6	2.27^b^	2.39^a^	0.06	0.004
18:2*n*-6	1.96^b^	2.05^a^	0.05	0.01
20:4*n*-6	0.12^b^	0.15^a^	0.01	<0.001
∑ *n*-6:*n*-3	4.82^b^	4.99^a^	0.01	0.04
∑ PUFA	2.74^b^	2.87^a^	0.07	0.01
∑ PUFA:SFA	0.05^b^	0.07^a^	0.01	<0.001
FA of microbial origin				
∑ BCFA	2.45^a^	2.32^b^	0.03	<0.001
*iso*-17:0	0.42^a^	0.40^b^	0.00	0.02
*anteiso-*17:0	0.63^b^	0.66^a^	0.01	<0.001
∑ *t*-18:1	7.28^a^	5.46^b^	0.15	<0.001
*t*11-18:1	3.35^a^	2.53^b^	0.07	<0.001
*t*13-*t*14	1.18^a^	0.76^b^	0.02	<0.001
∑ CLA	0.78^b^	1.29^a^	0.02	<0.001
*t*7,*c*9-18:2	0.04^b^	0.07^a^	0.00	<0.001
*c*9,*t*11-18:2	0.59^b^	1.06^a^	0.02	<0.001
*t*11,*c*13-18:2	0.03	0.03	0.00	0.30
∑ AD	0.57^b^	1.02^a^	0.02	<0.001
*t*8,*c*12-18:2	0.12^b^	0.24^a^	0.02	<0.001
*t*11,*c*15-18:2	0.17^b^	0.18^a^	0.02	0.001
∑ CLNA	0.09	0.09	0.00	0.62
*c*9,*t*11,*c*15-18:3	0.08^a^	0.07^b^	0.00	0.05

^a,b^Means with different superscripts for a particular fatty acid profile are significantly different (*P* <0.05); s.e.m, standard error of mean; *c*, *cis*; *t*, *trans*; ∑ FA, total fatty acids in mg/g of tissue; ∑ PUFA, sum of polyunsaturated fatty acids = ∑ *n*-6 + ∑ *n*-3; ∑ *n*-6 = sum of 18:2*n*-6, 20:3*n*-6, 20:4n-6; ∑ n-3 sum of 18:3n-3, 20:5n-3, 22:5n-3; ∑CLNA, sum of conjugated linolenic acid = *c*9,*t*11,*t*15-, *c*9,*t*11,*c*15-; ∑ AD, atypical dienes = sum of *t*11,*t*15-, *c*9,*t*13-/*t*8,*c*12-, *t*8,*c*13-, *c*9,*t*12-/*c*16-18:1, *t*9,*c*12-, *t*11,*c*15-, *c*9,*c*15-, *c*12,*c*15-18:2; ∑ CLA, conjugated linoleic acid = sum of *t*12,*t*14-, *t*11,*t*13-, *t*10,*t*12-, *t*9,*t*11-, *t*8,*t*10-, *t*7,*t*9- *t*6,*t*8-, *c*9,*t*11-, *t*7,*c*9-, *t*11,*c*13-, *t*12,*c*14-, *c*11,*t*13-, *t*10,*c*12-, *t*8,*c*10-, *t*9,*c*11-; ∑ *t*-MUFA, sum of trans-monounsaturated fatty acids = *t*9-16:1, *t*6,*t*7,*t*8-, *t*9-, *t*10-, *t*11-, *t*12-, *t*13/*t*14-, *t*15-, *t*16-18:1; ∑ *c*-MUFA = sum of *c*9-14:1, *c*7-16:1, *c*9-16:1, *c*11-16:1, *c*9-17:1, *c*9-18:1, *c*11-18:1, *c*12-18:1, *c*13-18:1, *c*14-18:1, *c*15-18:1, *c*9-20:1, *c*11-20:1; ∑ BCFA, branched chain fatty acids = sum of *iso-*15:0, *anteiso*-15:0, *iso*-16:0, *iso*-17:0, *anteiso-*17:0, *iso-*18:0; ∑ SFA = sum of 14:0, 15:0, 16:0, 17:0, 18:0, 19:0, 20:0.

Of the FA of dietary or endogenous origin, SFA and *cis*-monounsaturated FA (*c*-MUFA) were the major families (Table 2). Relative to hamburgers made with SCF, those made with PRF had greater (*P* <0.05) proportions of total and major SFA, with differences relating mostly to the higher proportions of 18:0 (Table 2). In support of the current findings, previous studies in beef cattle have shown that mature fat depots located internally such as PRF are more saturated than less mature fat depots located externally such as SCF [7, 27]. This has been attributed to a lower Δ-9 desaturase activity index in internal *vs.* external depots, and replacement of 18:0 with *c*9-18:1 in external fat depots.

Fat depot location had a significant effect on total *c*-MUFA, *c*9-14:1, *c*9-16:1 and *c*9-18:1, with SCF hamburgers having larger (*P* >0.05) proportions compared to PRF (Table 2). Current results agree with differences in Δ-9 desaturase gene expression (mRNA) reported by Lee et al. [27].

Fat depot location had no effect on total *n*-3 PUFA of hamburgers, however, there was a small but significant (*P* <0.05) increase in the proportion (0.01%) of docosanpentaenoic acid (22:5*n*-3) in SCF *vs.* PRF hamburgers (0.01%, *P* <0.05; Table 2). Total PUFA, total and major *n*-6 PUFA were influenced (*P* <0.05) by fat depot location, with SCF hamburgers having greater (*P* <0.05) proportions than PRF hamburgers.

#### Fatty acids of microbial origin

Fatty acids of microbial origin include branched-chain FA (BCFA) and PUFA-BHP which includes *trans*-monounsaturated FA (*t-*MUFA), CLA, non-conjugated 18:2 biohydrogenation products (*i.e.,* atypical dienes, AD) and conjugated linolenic acids ( CLNA). Hamburgers made with PRF *vs.* SCF had slightly greater (*P* <0.05) proportions of total BCFA, *iso*-17:0 and lower (*P* <0.05) proportions of *anteiso*-17:0. The reasons for these differences are not clear but could be linked to depot-specific differences in incorporation of individual FA [28].

Within PUFA-BHP, fat depot location influenced total and major *t*-18:1 (*t*11- and *t*13-/*t*14-18:1) isomers with proportions being greater (*P* <0.05) in PRF *vs.* SCF hamburgers (Table 2). In opposition, the proportions of total CLA, *t*7,*c*9-18:2 and *c*9,*t*11-18:2 were greater (*P* <0.05) in hamburgers made with SCF as opposed to PRF. Given *t*7,*c*9-18:2 and *c*9,*t*11-18:2 are known to be Δ-9 desaturase products of their respective *t*-18:1 precursors, these findings are consistent with the lower Δ-9 desaturase activity index reported for PRF compared to SCF [7, 27].

Total and major AD isomers were affected by fat depot location, with SCF having greater (*P* >0.05) proportions than PRF (Table 2). Fat depot location had no effect on total CLNA, but *c*9,*t*11,*c*15-18:3 proportions were slightly greater (*P* <0.05) in PRF than in SCF (Table 3). These findings could reflect differences between SCF and PRF in uptake or rate of metabolism of these FA [29].

**Table 3:** **Fatty acid profiles of hamburgers made with perirenal fat from steers fed a high forage diet containing sunflower-seed (SS) and 15 or 30% wheat dried distillers’ grains with solubles (DDGS)**

Items	Dietary treatments				s.e.m	***P*** -value
	Control	SS	DDGS-15	DDGS-30		
Variable	n = 16	n = 16	n = 16	n = 16		
∑ FA, mg/g	192^c^	186^d^	198^b^	202^a^	1	0.03
FA of endogenous or dietary origin						
∑ SFA	56.4^a^	55.8^b^	55.6^b^	54.4^c^	0.3	0.03
14:0	3.44^a^	2.90^b^	2.63^c^	2.90^b^	0.11	<0.001
16:0	27.0^a^	24.0^b^	23.4^c^	24.4^b^	0.1	<0.001
18:0	25.6^c^	28.5^a^	29.2^a^	26.7^b^	0.7	0.001
∑ *c*-MUFA	28.6^ab^	27.0^b^	26.9^b^	29.3^a^	0.6	0.02
*c*9-14:1	0.38^a^	0.31^b^	0.27^b^	0.35^a^	0.03	0.05
*c*9-16:1	1.68^a^	1.38^b^	1.27^b^	1.48^ab^	0.08	0.01
*c*9-18:1	23.9^ab^	22.5^c^	22.7^bc^	24.9^a^	0.5	0.003
∑ *n*-3	0.57^a^	0.45^b^	0.46^b^	0.41^c^	0.02	<0.001
18:3*n*-3	0.46^a^	0.38^b^	0.37^bc^	0.35^c^	0.01	<0.001
22:5*n*-3	0.11^a^	0.08^bc^	0.09^b^	0.07^c^	0.01	<0.001
∑ *n*-6	2.07^d^	2.16^c^	2.34^b^	2.50^a^	0.02	<0.001
18:2*n*-6	1.76^d^	1.88^c^	2.02^b^	2.20^a^	0.04	<0.001
20:4*n*-6	0.13	0.11	0.12	0.11	0.01	0.29
∑ *n*-6:*n*-3	3.63^d^	4.76^c^	5.12^b^	6.03^a^	0.03	0.04
∑ PUFA	2.64^b^	2.62^b^	2.79^ab^	2.92^a^	0.08	0.01
∑ PUFA:SFA	0.05	0.05	0.05	0.05	0.001	0.25
FA of microbial origin						
∑ BCFA	2.91^a^	2.42^b^	2.34^b^	2.14^c^	0.05	<0.001
*iso*-17:0	0.46^a^	0.41^b^	0.39^b^	0.36^c^	0.01	<0.001
*anteiso-*17:0	0.75^a^	0.60^b^	0.60^b^	0.55^c^	0.01	<0.001
∑ *t*-18:1	5.20^c^	8.11^a^	8.34^a^	7.48^b^	0.27	<0.001
*t*11-18:1	2.49^c^	3.69^a^	3.83^a^	3.39^b^	0.12	<0.001
*t*13-*t*14	0.83^c^	1.33^a^	1.35^a^	1.19^b^	0.05	<0.001
∑ CLA	0.69^b^	0.79^a^	0.82^a^	0.80^a^	0.03	0.001
*t*7,*c*9-18:2	0.03^b^	0.03^b^	0.04^a^	0.04^a^	0.00	<0.001
*c*9,*t*11-18:2	0.53^b^	0.61^a^	0.63^a^	0.62^a^	0.02	0.0005
*t*11,*c*13-18:2	0.03^a^	0.03^a^	0.03^a^	0.02^c^	0.00	<0.001
∑ AD	0.53^c^	0.57^b^	0.57^b^	0.60^a^	0.01	0.02
*t*8,*c*12-18:2	0.09^c^	0.12^b^	0.12^b^	0.14^a^	0.01	<0.001
*t*11,*c*15-18:2	0.19^a^	0.18^a^	0.16^b^	0.14^c^	0.01	<0.001
∑ CLNA	0.10	0.09	0.10	0.08	0.00	0.10
*c*9,*t*11,*c*15-18:3	0.08	0.08	0.08	0.07	0.00	0.20

^a,b,c,d^Means with different superscripts for a particular fatty acid profile are significantly different (*P* <0.05); s.e.m, standard error of mean; *c*, *cis*; *t*, *trans*; ∑ FA, total fatty acids in mg/g of tissue; ∑ PUFA, sum of polyunsaturated fatty acids = ∑ *n*-6 + ∑ *n*-3; ∑ *n*-6 = sum of 18:2*n*-6, 20:3*n*-6, 20:4n-6; ∑ n-3 sum of 18:3n-3, 20:5n-3, 22:5n-3; ∑CLNA, sum of conjugated linolenic acid = *c*9,*t*11,*t*15-, *c*9,*t*11,*c*15-; ∑ AD, atypical dienes = sum of *t*11,*t*15-, *c*9,*t*13-/*t*8,*c*12-, *t*8,*c*13-, *c*9,*t*12-/*c*16-18:1, *t*9,*c*12-, *t*11,*c*15-, *c*9,*c*15-, *c*12,*c*15-18:2; ∑ CLA, conjugated linoleic acid = sum of *t*12,*t*14-, *t*11,*t*13-, *t*10,*t*12-, *t*9,*t*11-, *t*8,*t*10-, *t*7,*t*9- *t*6,*t*8-, *c*9,*t*11-, *t*7,*c*9-, *t*11,*c*13-, *t*12,*c*14-, *c*11,*t*13-, *t*10,*c*12-, *t*8,*c*10-, *t*9,*c*11-; ∑ *t*-MUFA, sum of trans-monounsaturated fatty acids = *t*9-16:1, *t*6,*t*7,*t*8-, *t*9-, *t*10-, *t*11-, *t*12-, *t*13/*t*14-, *t*15-, *t*16-18:1; ∑ *c*-MUFA = sum of *c*9-14:1, *c*7-16:1, *c*9-16:1, *c*11-16:1, *c*9-17:1, *c*9-18:1, *c*11-18:1, *c*12-18:1, *c*13-18:1, *c*14-18:1, *c*15-18:1, *c*9-20:1, *c*11-20:1; ∑ BCFA, branched chain fatty acids = sum of *iso-*15:0, *anteiso*-15:0, *iso*-16:0, *iso*-17:0, *anteiso-*17:0, *iso-*18:0; ∑ SFA = sum of 14:0, 15:0, 16:0, 17:0, 18:0, 19:0, 20:0.

### Effects of animal diet on fatty acid composition of perirenal hamburgers

#### Fatty acids of endogenous or dietary origin

Substitution of SS for barley in the control diet reduced (*P* <0.05) the total FA content in hamburgers but feeding DDGS-15 and DDGS-30 led to successive increases (*P* <0.05; Table 3). These findings are consistent with differences in dietary energy levels, DMI, and potential differences in intramuscular fat contents [10]. Compared to control, feeding the SS diet reduced (*P* <0.05) total SFA, 14:0 and 16:0 (Table 3). Feeding the DDGS-15 diet led to no further change in total SFA (*P* >0.05) but further reduced (*P* >0.05) 14:0 and 16:0. Feeding the DDGS-30 diet further reduced (*P* <0.05) total SFA and increased (*P* <0.05) 14:0 and 16:0 back up to proportions equal to the SS diet. On the contrary, substitution of SS in the control diet increased (*P* <0.05) hamburger proportions of 18:0, feeding the DDGS-15 diet led to no further change (*P* >0.05), but feeding the DDGS-30 diet reduced 18:0 back down to proportions slightly above those found when feeding the control diet. These results resemble patterns of dietary proportions of the individual SFA observed in the current study, and may also relate to influences of both rates of complete biohydrogenation of PUFA to 18:0, and effects of higher proportions of 18:2*n*-6 in adipose tissues on *de novo* FA synthesis [30].

The proportions of total and individual *c*-MUFA declined (*P <*0.05) with substitution of SS in the control diet (Table 3). Feeding the DDGS-15 diet led to no further changes but feeding the DDGS-30 diet brought the proportions of these FA back up to proportions equal to the control diet (*P* >0.05). Differences in *c*-MUFA are difficult to interpret, as they did not follow trends in dietary proportions, and *c*-MUFA can originate from both diet and endogenous synthesis.

Substitution of SS in the control diet led to reductions (*P* <0.05) in the proportions of total and major *n*-3 PUFA in beef hamburgers and feeding the DDGS-15 and DDGS-30 diets led to further reductions (*P* <0.05; Table 3). This is consistent with the dietary proportions of 18:3*n*-3. Thus, these results could be related to the substitution of barley grain and red clover silage with SS and DDGS that have relatively lower concentrations of 18:3*n*-3. There is also a possibility that higher proportions of 18:2*n*-6 in the PRF of steers fed SS and DDGS diets might have reduced the elongation of 18:3*n*-3 to 22:5*n*-3 and/or 22:6*n-*3 due to competition for the Δ-6 desaturase [31]. The *n*-3 PUFA provide a wide range of benefits ranging from general improvements in health to protection against inflammation and disease [32] but the slight differences related to diet (>0.1%) may not be of practical significance.

Substitution of SS in the control diet increased the proportions of total PUFA, total *n*-6 PUFA and 18:2*n*-6, and feeding the DDGS-15 and DDGS-30 diets led to further increases (*P* <0.05; Table 3). These results reflect higher dietary proportions of 18:2*n*-6 observed in diets containing SS and potentially greater ruminal bypass of 18:2*n*-6 when feeding DDGS. On a practical basis, however, a difference between diets of <0.5% *n*-6 PUFA may not influence CVD risk.

Inclusion of SS and DDGS in the diet had no effect (*P* >0.05) on the PUFA:SFA ratio. The PUFA:SFA ratios for the hamburgers recorded in the current study were less than the lower limit recommended to improve human health [33], but again it can be difficult to recommend a ratio when individual FA within groups/families can have decidedly different biological effects.

#### Fatty acids of microbial origin

Substitution of SS in the control diet led to reductions (*P* >0.05) in the proportions of total and major BCFA, feeding the DDGS-15 diet resulted in no further changes (*P* <0.05), but feeding DDGS-30 resulted in further reductions (*P* >0.05). Since the majority of BCFA in animal tissue are synthesised *de novo* by rumen microbes [34], the high levels of 18:2*n*-6 in SS containing diets might have inhibited the rumen microbes responsible for BCFA production [35]. Further reductions observed when feeding the DDGS-30 diet could be a result of decreased ruminal propionate production from readily fermentable starch [36]. Propionate is a precursor of methylmalonate, which is utilised as a primer for the biosynthesis of BCFA [34]. *In vitro* and *in vivo* trials have shown that BCFA inhibit the growth of various cancer cell lines [37, 38]) and have demonstrated potential to reduce necrotizing enterocolitis in a neonatal rat model [39]. It would be of interest to determine if these limited changes (<0.8%) in BCFA would have any practical implications for human health.

Compared to control, proportions of total and major *t*-18:1 isomers were increased (*P* <0.05) by feeding the SS diet and feeding the DDGS-15 diet led to no further changes (*P* >0.05), while the increases were attenuated (*P* <0.05) when feeding the DDGS-30 diet (Table 3). This could be a result of a combination of factors including higher dietary 18:2*n*-6 observed for the diets containing SS, greater bypass of 18:2*n*-6 when feeding DDGS diets, and potential differences in ruminal pH which could have influenced PUFA biohydrogenation patterns [40, 41]. With *t*11-18:1 being associated with reductions in plasma triglycerides, total cholesterol and LDL-C in animal models [42, 43], and reductions in pro-inflammatory cytokines [44, 45] and platelet aggregation in humans [45] there has been a growing interest to increase its concentrations in beef. On the contrary, total *t*-18:1 FA and individual *t*-18:1 isomers other than *t*11-18:1 (*i.e., t*9- and *t*10-18:1) have been associated with increases in serum LDL-C and decreases in HDL-C in animal models [13, 46] but the effect of the remaining individual *t-*18:1 isomers on human health have not been investigated. Present results indicate that hamburgers made with PRF from steers fed SS and DDGS-15 diets could be enriched sources of potentially healthy *t*11-18:1. The increase in total *t*-MUFA was not, however, completely attributed to *t*11-18:1, and it would be important to evaluate the health effects of individual *t-*18:1 isomers, determine the levels regarded as beneficial or detrimental to human health, and develop feeding strategies that promote a healthier balance between isomers without negatively affecting animal performance and meat quality.

The proportions of total and major CLA isomers in beef hamburgers were increased (*P* <0.05) when substituting SS in the control diet but feeding the DDGS-15 and DDGS-30 diets led to no further changes (*P* >0.05; Table 3). Again, these findings could largely be ascribed to dissimilarities in dietary fibre content, 18:2*n*-6 proportions and bypass rates across diets. A minor diet effect was seen for *t*11*,c*13-18:2, with feeding the DDGS-30 diet yielding slightly lower (*P* <0.05) proportions compared to the other diets. The interest in raising the proportions of *c*9,*t*11-18:2 in beef is associated with its potential positive effects on human health [5]. In the present experiment, however, the increases in *c*9,*t*11-18:2 when feeding the SS, DDGS-15 and DDGS-30 diets were just over 0.1%, but it must be remembered effects on *c*9,*t*11-18:2 and *t*11-18:1 should be considered together given *t*11-18:1 is the precursor for *c*9,*t*11-18:2 in animals [5]. In addition, there is much to be understood regarding production of consistent amounts of *t*11-18:1 and *c*9,*t*11-18:2 across production cycles, as when feeding a diet very similar to the SS diet in the present experiment [8], the proportion of *t*11-18:1 in hamburger made with PRF would have been greater (5.9%) with a similar amount of *c*9,*t*11-18:2.

Compared to control, feeding the SS diet increased (*P* <0.05) proportions of total AD and *t*8,*c*12-18:2, the major AD likely largely derived from 18:2*n*-6, and no further changes were noted when feeding DDGS-15 (*P* <0.05) but feeding DDGS-30 led to further increases (*P* <0.05; Table 3). Overall, these findings reflect greater proportions of 18:2*n*-6 when feeding SS and DDGS diets. Substituting SS into the control diet had no effect on *t*11,*c*15-18:2, but feeding DDGS-15 and DDGS-30 led to reductions (*P* <0.05). This may be related to dietary proportions of *n*-3 PUFA which declined with additions of SS and DDGS to the diet. During biohydrogenation, 18:3*n*-3 is isomerised to CLNA, which is in turn hydrogenated to *t*11,*c*15-18:2 [47]. Feeding the SS, DDGS-15 and DDGS-30 diets had no effect (*P* <0.05) on total CLNA and *c*9,*t*11,*c*15-18:3, the major CLNA isomer (Table 3).

### Sensory attributes

#### Hamburgers made with perirenal vs. subcutaneous fat

Compared to SCF hamburgers, PRF hamburgers had higher (*P <*0.05) scores for initial and sustainable juiciness, tended to have higher scores for initial tenderness (*P* <0.09), but had lower (*P <*0.05) scores for beef flavour intensity (Table 4). Hamburgers made with PRF *vs.* SCF may have been juicier and tenderer due to their higher FA content and lower (*P* <0.05) cook loss (Table 4). The disparities in beef flavour intensity scores reported for PRF as opposed to SCF could be related to the differences in the proportions of *n*-6 and *n*-3 PUFA reported for these depots. Overall, oxidation of PUFA produces volatile compounds that may contribute to desirable or undesirable meat flavour depending on type, amounts and proportions in meat [48].

**Table 4:** **Sensory attributes of hamburgers made with perirenal or subcutaneous fat**

	Fat depot location		s.e.m	***P*** -value
Sensory attributes	Perirenal (n = 64)	Subcutaneous (n = 64)		
Initial tenderness	6.75	6.62	0.18	0.09
Overall tenderness	6.95	6.84	0.17	0.24
Initial juiciness	5.97^a^	5.43^b^	0.23	0.001
Sustainable juiciness	5.85^a^	5.63^b^	0.21	0.02
Beef flavour intensity	5.50^b^	5.67^a^	0.18	0.02
Off-flavour intensity	7.85	7.98	0.20	0.07
Residual mouth coating	6.04^b^	6.89^a^	0.34	0.001
Cooking attributes				
Cook loss, mg/gtissue	340^b^	382^a^	6.49	<0.001

^a,b^Means with different superscripts for a particular fatty acid profile are significantly different (*P* <0.05); s.e.m, standard error of mean; Attribute scores followed a nine point descriptive scale for initial and overall tenderness (9 = extremely tender; 1 = extremely tough), initial and sustainable juiciness (9 = extremely juicy; 1 = extremely dry), flavour and off-flavour intensity (9 = extremely intense; 1 = extremely bland/none), and residual mouth coating (9 = no mouth coating; 1 = extreme mouth coating).

Hamburgers made with PRF as opposed to SCF had lower (*P <*0.05) ratings for residual mouth coating, indicating greater coating (Table 4). This may be related to greater proportions of SFA, and 18:0 in particular, observed for the PRF depot. Higher proportions of 18:0 in meat fat would increase its melting point and consequently, the undesirable mouth-coating properties [49]. Although fat depot location influenced several sensory attributes of hamburgers, absolute differences were all less than one sensory panel unit, and would not likely be detected by untrained consumers who rarely consume plain hamburgers without added seasonings and/or condiments.

#### Dietary influence on hamburgers made with perirenal fat

Diet had no effect on the sensory attributes of hamburgers made with PRF except for residual mouth coating (Table 5). Feeding SS and DDGS-15 diets did not affect ratings for mouth-coating but slightly higher (*P <*0.05) ratings were observed when feeding the DDGS-30 diet (Table 5). The lower residual mouth coating reported for the DDGS-30 diet could be partly associated with lower SFA proportions observed for the same diet.

**Table 5:** **Sensory attributes of hamburgers made with perirenal fat from steers fed a high forage diet containing sunflower-seed (SS) and 15 or 30% wheat dried distillers’ grains with solubles (DDGS)**

	Dietary treatments				s.e.m	***P*** -value
Sensory attributes	Control	SS	DDGS-15	DDGS-30		
	n = 16	n = 16	n = 16	n = 16		
Initial tenderness	6.80	6.76	6.79	6.63	0.19	0.15
Overall tenderness	6.97	6.93	6.97	6.93	0.18	0.93
Initial juiciness	6.08	5.88	6.03	5.87	0.25	0.21
Sustainable juiciness	5.89	5.82	5.86	5.84	0.22	0.93
Beef flavour intensity	5.46	5.48	5.47	5.59	0.20	0.62
Off-flavour intensity	7.74	7.93	7.82	7.96	0.24	0.51
Residual mouth coating	5.96^b^	5.95^b^	6.00^b^	6.32^a^	0.32	0.02
Cooking attributes						
Cook loss, mg/g tissue	334	343	332	352	8.52	0.17

^a**,**b^Means with different superscripts for a particular fatty acid profile are significantly different (*P* <0.05); s.e.m, standard error of mean; Attribute scores followed a nine point descriptive scale for initial and overall tenderness (9 = extremely tender; 1 = extremely tough), initial and sustainable juiciness (9 = extremely juicy; 1 = extremely dry), flavour and off-flavour intensity (9 = extremely intense; 1 = extremely bland/none), and residual mouth coating (9 = no mouth coating; 1 = extreme mouth coating).

### Changes in oxidative stability of hamburgers during retail display

#### Perirenal vs. subcutaneous hamburgers

Over four days of retail display, hamburgers made with either PRF or SCF had substantial increases (*P* <0.05) in hue angle (yellowing) combined with reductions (*P* <0.05) in chroma (colour intensity) and L* (lightness). In addition, during retail display metmyoglobin and malonaldehyde increased (*P* <0.05) while oxymyoglobin decreased (*P* <0.05). Effects of fat depot location on hamburger changes during retail display were, however, limited to a slight but greater reduction (*P* <0.05) in L* and a trend for less of a reduction (*P* = 0.07) in chroma when making hamburgers with SCF (Table 6). This may be due to the fact that SCF contain more water than PRF [26].

**Table 6:** **Change in retail colour and oxidative stability of hamburgers made with perirenal or subcutaneous fat over 4 d**

	Fat depot location			
Objective retail measurements	Perirenal (n = 64)	Subcutaneous (n = 64)	s.e.m	***P*** -value
L*	-1.28^b^	-1.87^a^	0.11	0.04
Chroma	-4.23	-3.88	0.34	0.07
Hue	12.1	10.9	0.4	0.15
Metmyoglobin	0.15	0.15	-0.36	0.18
Oxymyoglobin	-0.19	-0.19	0.59	0.99
Malonaldehyde, mg/kg of meat	0.16	0.16	0.04	0.95

^a**,**b^Means with different superscripts for a particular fatty acid profile are significantly different (*P* <0.05); s.e.m, standard error of mean.

L* refers to the light-dark axis for the 3 dimensional colour space (i.e. the value describes the lightness of the colour).

#### Dietary influence on perirenal hamburgers

Feeding SS or DGGS diets neither changed (*P <*0.05) colour (L*, hue or chroma) nor concentrations of oxymyoglobin, metmyoglobin and malonaldehyde of PRF hamburgers during retail display.

## Conclusions

The most notable differences between the two fat depots were those related to the greater proportions of SFA, chiefly 18:0, *t*-18:1 isomers, primarily *t*11-18:1 and BCFA in PRF. Feeding SS and DDGS-15 diets compared to the control diet led to increases in proportions of *t*11-18:1 and *c*9,*t*11-18:2 in PRF, but feeding DDGS-30 was less effective. Feeding the DDGS-15 diet might, therefore, be a way to improve the healthfulness of perirenal FA profiles, while improving overall animal performance and meat quality.
